# Ethyl 4-methyl-1,3-dioxo-1,2,3,4-tetra­hydro­isoquinoline-4-carboxyl­ate

**DOI:** 10.1107/S1600536812008136

**Published:** 2012-02-29

**Authors:** Xing-Yao Li, Jin-Long Wu

**Affiliations:** a Laboratory of Asymmetric Catalysis and Synthesis, Department of Chemistry, Zhejiang University, Hangzhou 310027, People’s Republic of China

## Abstract

In the title compound, C_13_H_13_NO_4_, the fused-ring system is nearly planar, with an r.m.s. deviation of 0.0408 Å. In the crystal, mol­ecules are linked into centrosymmetric dimers by a pair of N—H⋯O hydrogen bonds. The ethyl group is disordered over two positions in a ratio of 0.758 (6):0.242 (6).

## Related literature

For pharmaceutical usage of derivatives of isoquinoline-1,3(2*H*,4*H*)-dione, see: Lu *et al.* (2010 ▶); Tsou *et al.* (2008 ▶, 2009 ▶); Billamboz *et al.* (2011 ▶).
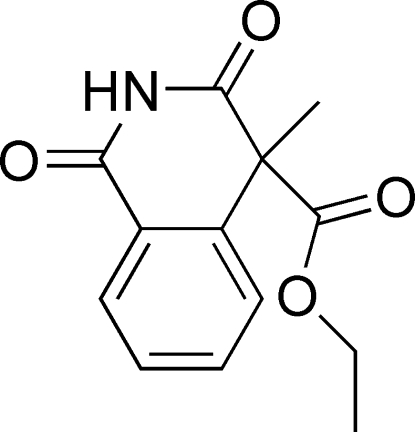


## Experimental

### 

#### Crystal data

C_13_H_13_NO_4_
*M* *_r_* = 247.24
Triclinic, 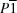

*a* = 6.4585 (9) Å
*b* = 8.1999 (7) Å
*c* = 12.5763 (11) Å
α = 78.876 (7)°
β = 77.228 (9)°
γ = 72.354 (9)°
*V* = 613.28 (11) Å^3^
*Z* = 2
Mo *K*α radiation
μ = 0.10 mm^−1^
*T* = 293 K
0.38 × 0.23 × 0.09 mm

#### Data collection

Agilent Xcalibur Atlas Gemini ultra diffractometer
Absorption correction: multi-scan (*CrysAlis PRO*; Agilent, 2010 ▶) *T*_min_ = 0.963, *T*_max_ = 0.991
3820 measured reflections
2243 independent reflections
1659 reflections with *I* > 2σ(*I*)
*R*_int_ = 0.021

#### Refinement

*R*[*F* ^2^ > 2σ(*F* ^2^)] = 0.049
*wR*(*F* ^2^) = 0.135
*S* = 1.06
2243 reflections
170 parameters
5 restraints
H-atom parameters constrained
Δρ_max_ = 0.18 e Å^−3^
Δρ_min_ = −0.25 e Å^−3^

### 

Data collection: *CrysAlis PRO* (Agilent, 2010 ▶); cell refinement: *CrysAlis PRO*; data reduction: *CrysAlis PRO*; program(s) used to solve structure: *SHELXS97* (Sheldrick, 2008 ▶); program(s) used to refine structure: *SHELXL97* (Sheldrick, 2008 ▶); molecular graphics: *OLEX2* (Dolomanov *et al.*, 2009 ▶); software used to prepare material for publication: *OLEX2*.

## Supplementary Material

Crystal structure: contains datablock(s) I, global. DOI: 10.1107/S1600536812008136/xu5472sup1.cif

Structure factors: contains datablock(s) I. DOI: 10.1107/S1600536812008136/xu5472Isup2.hkl

Supplementary material file. DOI: 10.1107/S1600536812008136/xu5472Isup3.cml

Additional supplementary materials:  crystallographic information; 3D view; checkCIF report

## Figures and Tables

**Table 1:** Hydrogen-bond geometry (Å, °)

*D*—H⋯*A*	*D*—H	H⋯*A*	*D*⋯*A*	*D*—H⋯*A*
N1—H1⋯O1^i^	0.86	2.05	2.903 (3)	172

Symmetry code: (i) 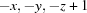.

**Fig. 1.**
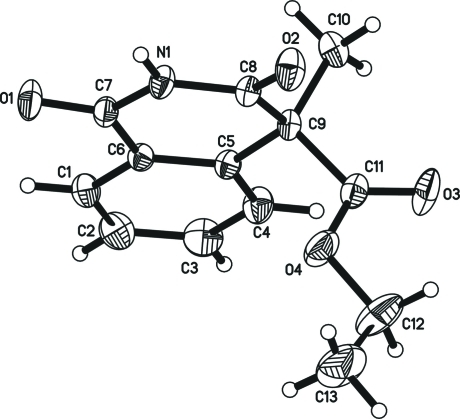
The molecular structure of the title compound with displacement ellipsoids are drawn at 30% probability level. H atoms are presented as a small spheres of arbitrary radius.

**Fig. 2.**
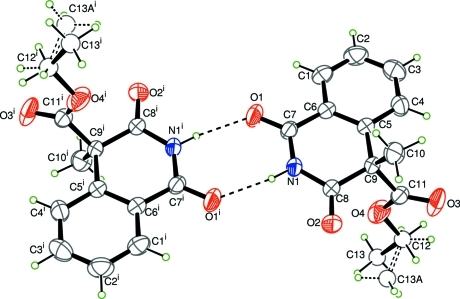
Centrosymmetric dimers of the title compound linked by two N—H···O hydrogen bonds (dotted lines). Symmetry code: (i) -x, -y, -z+1.

## supplementary crystallographic information

### Comment

Derivatives of isoquinoline-1,3(2H,4H)-dione are important compounds in pharmaceutical chemistry and have great research values, such as inhibitors of the cyclic-dependent kinase 4 (CDK4) (Tsou *et al.*, 2008, 2009; Lu *et al.*., 2010); inhibitors of HIV-1 integrase (Billamboz *et al.*, 2011). In our research of synthesis of cyclonitrones, we have obtained the title compound as a minor product from ethyl 2-(2-(1,3-dioxolan-2-yl)phenyl)-2-cyanopropanoate hydrolysed by hydrogen peroxide. The structure of the title compound has been characterized by spectroscopic methods and further confirmation by X-ray analysis. We report here its crystal structure. In the molecule of the title compound (Fig. 1), there is one benzene ring fused by carbonyl amide closing six-membered heterocyclic ring, the two rings are almost coplanar with only 1.02 (10)° dihedral angle. One sterogenic center but the crystallizes as a racemate as indicated by the centrosymmetric space group. In the crystal structure, molecules are linked by two N—H···O hydrogen bonds into dimers that are located on centres of inversion.

### Experimental

At room temperature, to a solution of ethyl 2-(2-(1,3-dioxolan-2-yl) phenyl)-2-cyanopropanoate (344 mg, 1.25 mmol) in DMSO (3.0 ml) was added K_2_CO_3_ (89 mg, 0.64 eq), after then H_2_O_2_ (0.55 mL, 30%, 4 eq) was added as three portions in 2 hours. The mixture was then stirred for another 1h and quenched with brine (20 mL). The resulting mixture was subjected to extraction with ethyl acetate (2 x 30 mL). The combined organic phase was washed with brine (2 x 20 mL), dried over Na_2_SO_4_, concentrated in vacuo, and the residue was subjected to flash chromatography (silica gel, 25% ethyl acetate in hexane) to give ethyl 2-(2-(1,3-dioxolan-2-yl)phenyl)-3-amino-2-methyl-3-oxopropanoate (264 mg, 72%) as a colorless solid and the title compound, ethyl 4-methyl-1,3-dioxo-1,2,3,4-tetrahydroisoquinoline-4-carboxylate (50 mg, 16%) as colorless needles (m.p. 440-441.5 K). Single crystals suitable for X-ray diffraction of the title compound were grown at ambient temperature in dichloromethane.

### Refinement

The H atoms were placed in calculated positions with C—H = 0.93–0.97 Å, N—H = 0.86 Å and included in the refinement as riding their carrier atoms with *U*_iso_(H)=1.2*U*_eq_(C,N).

### Crystal data

**Table:**

C_13_H_13_NO_4_	*Z* = 2
*M**_r_* = 247.24	*F*(000) = 260
Triclinic, *P*1	*D*_x_ = 1.339 Mg m^−^^3^
Hall symbol: -P 1	Mo *K*α radiation, λ = 0.71073 Å
*a* = 6.4585 (9) Å	Cell parameters from 1387 reflections
*b* = 8.1999 (7) Å	θ = 3.3–29.2°
*c* = 12.5763 (11) Å	µ = 0.10 mm^−^^1^
α = 78.876 (7)°	*T* = 293 K
β = 77.228 (9)°	Platelet, colorless
γ = 72.354 (9)°	0.38 × 0.23 × 0.09 mm
*V* = 613.28 (11) Å^3^	

### Data collection

**Table:**

Agilent Xcalibur Atlas Gemini ultra diffractometer	2243 independent reflections
Radiation source: fine-focus sealed tube	1659 reflections with *I* > 2σ(*I*)
Graphite monochromator	*R*_int_ = 0.021
Detector resolution: 10.3592 pixels mm^-1^	θ_max_ = 25.4°, θ_min_ = 3.3°
ω scans	*h* = −7→6
Absorption correction: multi-scan (*CrysAlis PRO*; Agilent, 2010)	*k* = −9→9
*T*_min_ = 0.963, *T*_max_ = 0.991	*l* = −14→15
3820 measured reflections	

### Refinement

**Table:**

Refinement on *F*^2^	Primary atom site location: structure-invariant direct methods
Least-squares matrix: full	Secondary atom site location: difference Fourier map
*R*[*F*^2^ > 2σ(*F*^2^)] = 0.049	Hydrogen site location: inferred from neighbouring sites
*wR*(*F*^2^) = 0.135	H-atom parameters constrained
*S* = 1.06	*w* = 1/[σ^2^(*F*_o_^2^) + (0.061*P*)^2^ + 0.1013*P*] where *P* = (*F*_o_^2^ + 2*F*_c_^2^)/3
2243 reflections	(Δ/σ)_max_ < 0.001
170 parameters	Δρ_max_ = 0.18 e Å^−^^3^
5 restraints	Δρ_min_ = −0.25 e Å^−^^3^

### Special details

**Table:**

Experimental. ^1^H NMR (400 MHz, CDCl_3_): 8.37 (br s, 1H), 8.26 (d, *J* = 8.0 Hz, 1H), 7.68 (t, *J* = 8.0 Hz, 1H), 7.52 (t, *J* = 8.0 Hz, 1H), 7.41 (d, *J* = 7.6 Hz, 1H), 4.23-4.05 (m, 2H), 1.89 (s, 3H), 1.12 (t, *J* = 7.2 Hz, 3H) ppm; ^13^C NMR (100 MHz, CDCl_3_): 171.1, 169.1, 163.6, 140.1, 134.8, 128.9, 128.6, 125.9, 123.5, 62.6, 55.0, 25.1, 13.7 ppm.
Geometry. All esds (except the esd in the dihedral angle between two l.s. planes) are estimated using the full covariance matrix. The cell esds are taken into account individually in the estimation of esds in distances, angles and torsion angles; correlations between esds in cell parameters are only used when they are defined by crystal symmetry. An approximate (isotropic) treatment of cell esds is used for estimating esds involving l.s. planes.
Refinement. Refinement of F^2^ against ALL reflections. The weighted R-factor wR and goodness of fit S are based on F^2^, conventional R-factors R are based on F, with F set to zero for negative F^2^. The threshold expression of F^2^ > 2sigma(F^2^) is used only for calculating R-factors(gt) etc. and is not relevant to the choice of reflections for refinement. R-factors based on F^2^ are statistically about twice as large as those based on F, and R- factors based on ALL data will be even larger.

### Fractional atomic coordinates and isotropic or equivalent isotropic displacement parameters (Å^2^)

**Table:**

	*x*	*y*	*z*	*U*_iso_*/*U*_eq_	Occ. (<1)
O1	0.2337 (3)	−0.13049 (17)	0.54786 (12)	0.0529 (4)	
O2	−0.1215 (2)	0.40108 (18)	0.63638 (13)	0.0527 (4)	
O3	0.1682 (3)	0.4585 (2)	0.83716 (14)	0.0734 (6)	
O4	−0.0080 (3)	0.2543 (2)	0.86413 (12)	0.0610 (5)	
N1	0.0689 (3)	0.13902 (19)	0.58943 (13)	0.0379 (4)	
H1	−0.0268	0.1471	0.5494	0.046*	
C1	0.5645 (4)	−0.1829 (3)	0.67306 (17)	0.0453 (5)	
H1A	0.5636	−0.2759	0.6411	0.054*	
C2	0.7280 (4)	−0.1987 (3)	0.7311 (2)	0.0559 (6)	
H2	0.8373	−0.3023	0.7390	0.067*	
C3	0.7288 (4)	−0.0596 (3)	0.7776 (2)	0.0578 (6)	
H3	0.8405	−0.0696	0.8161	0.069*	
C4	0.5663 (4)	0.0937 (3)	0.76769 (18)	0.0477 (6)	
H4	0.5684	0.1858	0.8001	0.057*	
C5	0.3988 (3)	0.1116 (2)	0.70940 (15)	0.0333 (4)	
C6	0.4003 (3)	−0.0284 (2)	0.66182 (14)	0.0333 (4)	
C7	0.2317 (3)	−0.0136 (2)	0.59585 (15)	0.0353 (5)	
C8	0.0413 (3)	0.2804 (2)	0.63970 (15)	0.0338 (4)	
C9	0.2282 (3)	0.2843 (2)	0.69348 (14)	0.0325 (4)	
C10	0.3389 (4)	0.4169 (3)	0.61867 (18)	0.0478 (5)	
H10A	0.4522	0.4298	0.6520	0.072*	
H10B	0.2307	0.5261	0.6088	0.072*	
H10C	0.4030	0.3776	0.5485	0.072*	
C11	0.1267 (3)	0.3452 (3)	0.80564 (17)	0.0420 (5)	
C12	−0.1109 (6)	0.3031 (5)	0.9735 (2)	0.0928 (10)	
H12A	0.0009	0.2758	1.0194	0.111*	0.758 (6)
H12B	−0.1798	0.4266	0.9674	0.111*	0.758 (6)
H12C	−0.0798	0.2026	1.0286	0.111*	0.242 (6)
H12D	−0.0493	0.3888	0.9884	0.111*	0.242 (6)
C13	−0.2713 (9)	0.2137 (8)	1.0226 (3)	0.1076 (18)	0.758 (6)
H13A	−0.3858	0.2455	0.9791	0.161*	0.758 (6)
H13B	−0.3334	0.2432	1.0954	0.161*	0.758 (6)
H13C	−0.2036	0.0915	1.0268	0.161*	0.758 (6)
C13A	−0.340 (2)	0.371 (2)	0.9802 (11)	0.1076 (18)	0.242 (6)
H13D	−0.3722	0.4842	0.9389	0.161*	0.242 (6)
H13E	−0.4090	0.3774	1.0558	0.161*	0.242 (6)
H13F	−0.3969	0.2969	0.9504	0.161*	0.242 (6)

### Atomic displacement parameters (Å^2^)

**Table:**

	*U*^11^	*U*^22^	*U*^33^	*U*^12^	*U*^13^	*U*^23^
O1	0.0628 (10)	0.0400 (8)	0.0648 (10)	−0.0090 (7)	−0.0201 (8)	−0.0254 (7)
O2	0.0404 (9)	0.0454 (9)	0.0740 (10)	0.0051 (7)	−0.0223 (7)	−0.0247 (8)
O3	0.0921 (14)	0.0731 (11)	0.0728 (11)	−0.0273 (10)	−0.0124 (10)	−0.0462 (10)
O4	0.0681 (11)	0.0740 (11)	0.0415 (8)	−0.0234 (9)	0.0101 (7)	−0.0258 (8)
N1	0.0375 (10)	0.0385 (9)	0.0429 (9)	−0.0059 (7)	−0.0146 (7)	−0.0159 (7)
C1	0.0484 (13)	0.0332 (11)	0.0496 (12)	−0.0030 (9)	−0.0069 (10)	−0.0094 (9)
C2	0.0492 (14)	0.0422 (12)	0.0656 (15)	0.0057 (10)	−0.0151 (11)	−0.0057 (11)
C3	0.0463 (14)	0.0610 (15)	0.0650 (15)	−0.0035 (12)	−0.0275 (11)	−0.0035 (12)
C4	0.0460 (13)	0.0464 (12)	0.0569 (13)	−0.0082 (10)	−0.0224 (10)	−0.0133 (10)
C5	0.0323 (10)	0.0331 (10)	0.0342 (10)	−0.0069 (8)	−0.0043 (8)	−0.0089 (8)
C6	0.0351 (11)	0.0307 (10)	0.0323 (10)	−0.0078 (8)	−0.0024 (8)	−0.0062 (8)
C7	0.0385 (11)	0.0320 (10)	0.0371 (10)	−0.0104 (9)	−0.0027 (8)	−0.0114 (8)
C8	0.0323 (11)	0.0333 (10)	0.0367 (10)	−0.0072 (8)	−0.0050 (8)	−0.0112 (8)
C9	0.0329 (10)	0.0293 (9)	0.0380 (10)	−0.0073 (8)	−0.0070 (8)	−0.0116 (8)
C10	0.0494 (13)	0.0361 (11)	0.0607 (13)	−0.0152 (10)	−0.0107 (10)	−0.0058 (10)
C11	0.0405 (12)	0.0409 (11)	0.0459 (12)	−0.0019 (10)	−0.0133 (9)	−0.0168 (10)
C12	0.094 (2)	0.134 (3)	0.0461 (15)	−0.029 (2)	0.0168 (14)	−0.0391 (17)
C13	0.125 (4)	0.143 (5)	0.059 (3)	−0.068 (4)	0.035 (2)	−0.030 (3)
C13A	0.125 (4)	0.143 (5)	0.059 (3)	−0.068 (4)	0.035 (2)	−0.030 (3)

### Geometric parameters (Å, º)

**Table:**

O1—C7	1.223 (2)	C6—C7	1.473 (3)
O2—C8	1.208 (2)	C8—C9	1.519 (3)
O3—C11	1.197 (2)	C9—C11	1.527 (3)
O4—C11	1.324 (3)	C9—C10	1.535 (3)
O4—C12	1.463 (3)	C10—H10A	0.9600
N1—C7	1.371 (2)	C10—H10B	0.9600
N1—C8	1.372 (2)	C10—H10C	0.9600
N1—H1	0.8600	C12—C13A	1.406 (13)
C1—C2	1.373 (3)	C12—C13	1.412 (5)
C1—C6	1.390 (3)	C12—H12A	0.9700
C1—H1A	0.9300	C12—H12B	0.9700
C2—C3	1.380 (3)	C12—H12C	0.9700
C2—H2	0.9300	C12—H12D	0.9700
C3—C4	1.376 (3)	C13—H13A	0.9600
C3—H3	0.9300	C13—H13B	0.9600
C4—C5	1.394 (3)	C13—H13C	0.9600
C4—H4	0.9300	C13A—H13D	0.9600
C5—C6	1.390 (2)	C13A—H13E	0.9600
C5—C9	1.517 (2)	C13A—H13F	0.9600
			
C11—O4—C12	115.2 (2)	C9—C10—H10C	109.5
C7—N1—C8	127.34 (15)	H10A—C10—H10C	109.5
C7—N1—H1	116.3	H10B—C10—H10C	109.5
C8—N1—H1	116.3	O3—C11—O4	124.5 (2)
C2—C1—C6	120.26 (19)	O3—C11—C9	123.9 (2)
C2—C1—H1A	119.9	O4—C11—C9	111.54 (16)
C6—C1—H1A	119.9	C13A—C12—C13	54.0 (7)
C1—C2—C3	119.5 (2)	C13A—C12—O4	110.5 (6)
C1—C2—H2	120.3	C13—C12—O4	110.2 (3)
C3—C2—H2	120.3	C13A—C12—H12A	139.8
C4—C3—C2	120.8 (2)	C13—C12—H12A	109.6
C4—C3—H3	119.6	O4—C12—H12A	109.6
C2—C3—H3	119.6	C13A—C12—H12B	58.6
C3—C4—C5	120.40 (19)	C13—C12—H12B	109.6
C3—C4—H4	119.8	O4—C12—H12B	109.6
C5—C4—H4	119.8	H12A—C12—H12B	108.1
C6—C5—C4	118.45 (18)	C13A—C12—H12C	109.5
C6—C5—C9	121.52 (16)	C13—C12—H12C	58.8
C4—C5—C9	119.93 (16)	O4—C12—H12C	109.5
C1—C6—C5	120.58 (18)	H12A—C12—H12C	54.2
C1—C6—C7	118.94 (17)	H12B—C12—H12C	140.7
C5—C6—C7	120.46 (16)	C13A—C12—H12D	109.5
O1—C7—N1	120.24 (17)	C13—C12—H12D	140.2
O1—C7—C6	122.58 (17)	O4—C12—H12D	109.5
N1—C7—C6	117.17 (15)	H12A—C12—H12D	56.7
O2—C8—N1	120.90 (17)	H12B—C12—H12D	54.3
O2—C8—C9	121.31 (15)	H12C—C12—H12D	108.1
N1—C8—C9	117.70 (15)	C12—C13—H13A	109.5
C5—C9—C8	114.29 (15)	C12—C13—H13B	109.5
C5—C9—C11	108.88 (15)	C12—C13—H13C	109.5
C8—C9—C11	107.75 (15)	C12—C13A—H13D	109.5
C5—C9—C10	109.59 (16)	C12—C13A—H13E	109.5
C8—C9—C10	106.40 (15)	H13D—C13A—H13E	109.5
C11—C9—C10	109.85 (16)	C12—C13A—H13F	109.5
C9—C10—H10A	109.5	H13D—C13A—H13F	109.5
C9—C10—H10B	109.5	H13E—C13A—H13F	109.5
H10A—C10—H10B	109.5		

### Hydrogen-bond geometry (Å, º)

**Table:**

*D*—H···*A*	*D*—H	H···*A*	*D*···*A*	*D*—H···*A*
N1—H1···O1^i^	0.86	2.05	2.903 (3)	172

Symmetry code: (i) −*x*, −*y*, −*z*+1.

## References

[bb1] Agilent (2010). *CrysAlis PRO* Agilent Technologies, Yarnton, Oxfordshire, England.

[bb2] Billamboz, M., Bailly, F., Lion, C., Touati, N., Vezin, H., Calmels, C., Andreola, M.-L., Christ, F., Debyser, Z. & Cotelle, P. (2011). *J. Med. Chem.* **54**, 1812-1824. 10.1021/jm1014692 21366258

[bb3] Dolomanov, O. V., Bourhis, L. J., Gildea, R. J., Howard, J. A. K. & Puschmann, H. (2009). *J. Appl. Cryst.* **42**, 339–341.

[bb4] Lu, X.-Y., Chen, Y.-D., Sun, N.-Y., Jiang, Y.-J. & You, Q.-D. (2010). *J. Mol. Model.* **16**, 163-173. 10.1007/s00894-009-0529-7 19543928

[bb5] Sheldrick, G. M. (2008). *Acta Cryst.* A**64**, 112–122. 10.1107/S0108767307043930 18156677

[bb6] Tsou, H. R., Liu, X.-X., Birnberg, G., Kaplan, J., Otteng, M., Tran, T., Kutterer, K., Tang, Z.-L., Suayan, R., Zask, A., Ravi, M., Bretz, A., Grillo, M., McGinnis, J. P., Rabindran, S. K., Ayral-Kaloustian, S. & Mansour, T. S. (2009). *J. Med. Chem.* **52**, 2289-2310. 10.1021/jm801026e 19317452

[bb7] Tsou, H. R., Otteng, M., Tran, T., Floyd, M. B. Jr, Reich, M., Birnberg, G., Kutterer, K., Ayral-Kaloustian, S., Ravi, M., Nilakantan, R., Grillo, M., McGinnis, J. P. & Rabindran, S. K. (2008). *J. Med. Chem.* **51**, 3507-3525. 10.1021/jm800072z 18494457

